# Induction of Biosynthesis Antioxidant Molecules in Young Barley Plants by Trioxygen

**DOI:** 10.3390/molecules27217195

**Published:** 2022-10-24

**Authors:** Natalia Matłok, Tomasz Piechowiak, Ireneusz Kapusta, Kamil Królikowski, Maciej Balawejder

**Affiliations:** 1 Department of Food and Agriculture Production Engineering, University of Rzeszow, St. Zelwerowicza 4, 35-601 Rzeszow, Poland; 2 Department of Chemistry and Food Toxicology, University of Rzeszow, St. Ćwiklińskiej 1a, 35-601 Rzeszow, Poland; 3 Department of Food Technology and Human Nutrition, Rzeszow University, St. Zelwerowicza 4, 35-601 Rzeszow, Poland; 4 College of Natural Sciences, University of Rzeszow, St. Zelwerowicza 4, 35-601 Rzeszow, Poland

**Keywords:** barley young leaves, antioxidant activities, polyphenols, UPLC–PDA–MS/MS, saponarin (isovitexin 7-*O*-glucoside), ROS generation, SOD activity, COT activity, GPOX activity, PAL activity, PPO activity

## Abstract

Young barley plants are a good source of bioactive compounds. This paper presents the effects of gaseous O_3_ (trioxygen or ozone) on the biosynthesis of compounds, determining the antioxidant potential of young barley plants. The total content of polyphenols was determined along with their profile, as well as total antioxidant potential and vitamin C content. The highest contents of these compounds were identified in young barley plants exposed to gaseous O_3_. The main bioactive compound, representing polyphenols, determined in the examined raw materials was saponarin (isovitexin 7-*O*-glucoside). The induction of increased biosynthesis of these molecules was directly linked to the modification of the activity of selected enzymes. The increased polyphenol content resulted from the modified activities of polyphenol oxidase (PPO) and phenylalanine ammonia lyase (PAL). On the other hand, the oxidative effect of ozone on barley plants was reduced, owing to the modified activities of catalases (CAT), glutathione peroxidases (SOD) and guaiacol peroxidase (GPOX). Analysis of the results showed that by applying gaseous O_3_ at a dose of 50 ppm for 10 min, the contents of bioactive compounds can be maximised in a residue-free way by activating oxidative stress defence mechanisms.

## 1. Introduction

The dietary trends promoted today directly correspond to research findings showing a relationship between nutrition and severe disorders, most importantly cancer and cardiovascular diseases, which contribute to the need for natural raw materials containing bioactive compounds.

Comprehensively investigated groups of bioactive compounds include polyphenols, whose biological activity has been demonstrated by in vitro and in vivo studies. A number of studies suggest that these substances exhibit antineoplastic and other biological activity, reflected by, e.g., neuroprotective, anti-allergic and anti-aggregative effects [1]. For instance, regular consumption of flavonoids was shown to significantly reduce the incidence of neoplastic diseases, including breast cancer [2,3], whereas preclinical research presented evidence that flavonoids inhibited the in vivo and in vitro development of mammary cancer [4,5].

At the early stages of their development, plants often generate increased amounts of bioactive compounds. This has been observed, for instance, in barley (*Hordeum vulgare* L.) seedlings. Plants of this species are most commonly used at the growth stage in which leaves are 10–15 cm long [6]. The examination of such plants using liquid chromatography with a tandem mass spectrometer identified the contents of 37 polyphenols, mainly flavonoids and hydroxycinnamic acid derivatives [7]. The main polyphenolic compounds contained in young barley plants are saponarin (isovitexin-7-*O*-glucoside) and 2-*O*-glycosyl isovitexin [8]. Saponarin exhibits anti-inflammatory and anti-allergic properties; it has also been hypothesised that the compound has anti-neoplastic properties, but this has not been confirmed by in vivo studies [9].

Young barley is also a source of other small-molecule antioxidants and vitamins (C, E, thiamine (B1), riboflavin (B2), pyridoxine (B6), pantothenic acid (B5), folic acid (B9)), as well as β-carotene and chlorophyll. It is also a good source of easily available minerals such as calcium, copper, iron, magnesium, potassium and zinc [10,11,12,13].

The contents of bioactive compounds in plant materials can be modified by altering soil and climate conditions or by applying appropriate fertilisation [14,15]. The activity of other factors, such as UV radiation [16] or electromagnetic fields [17], and by altering the atmosphere, for instance with the gaseous ozone (O_3_), were also validated [18]. The latter is a promising method for modifying the contents of bioactive compounds in raw plant materials since gaseous O_3_ does not generate residues. Ozone treatment correctly applied to raw plant materials (fruit, herbs and other crops) has been reported to increase the content of bioactive compounds [19,20,21,22]. These observations are extremely important given the fact that negative effects of chronic exposure of plants to gaseous O_3_ have predominantly been reported by the related research [23].

The study aimed to induce the biosynthesis of compounds affecting the antioxidant potential of young barley plants by applying gaseous trioxygen (O_3_). The assessments were performed to determine the effects of the dose of O_3_ applied on the total content of polyphenols, their profile, as well as total antioxidant potential and vitamin C contents; additionally, the mechanism of the effects produced by gaseous O_3_ was investigated by measuring the activity of selected enzymes, oxidative stress markers and levels of reactive oxygen species (ROS).

## 2. Results and Discussion

### 2.1. Antioxidant Activity, Total Phenolic Content and Ascorbic Acid Content

**Figure 1:**
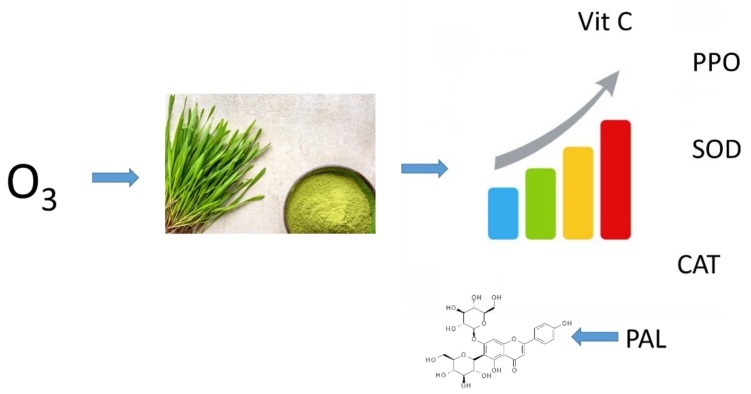
Proposed mechanism of trioxygen induced biosynthesis antioxidant molecules in young barley.

Young barley plants are a good source of polyphenols, whose content depends on the conditions existing during the plant growth. By applying certain crop enhancement methods, including various fertilisers, it is possible to slightly modify the contents of these compounds. However, as a result of such treatments, the plant material produced contains residues from the applied fertilisers [24]. Gaseous ozone can be used to modify plant metabolism in a residue-free way and, consequently, to enhance the raw material produced by increasing the content of non-nutritive components, mainly small-molecule antioxidants (Figure 1) [25].

**Figure 2:**
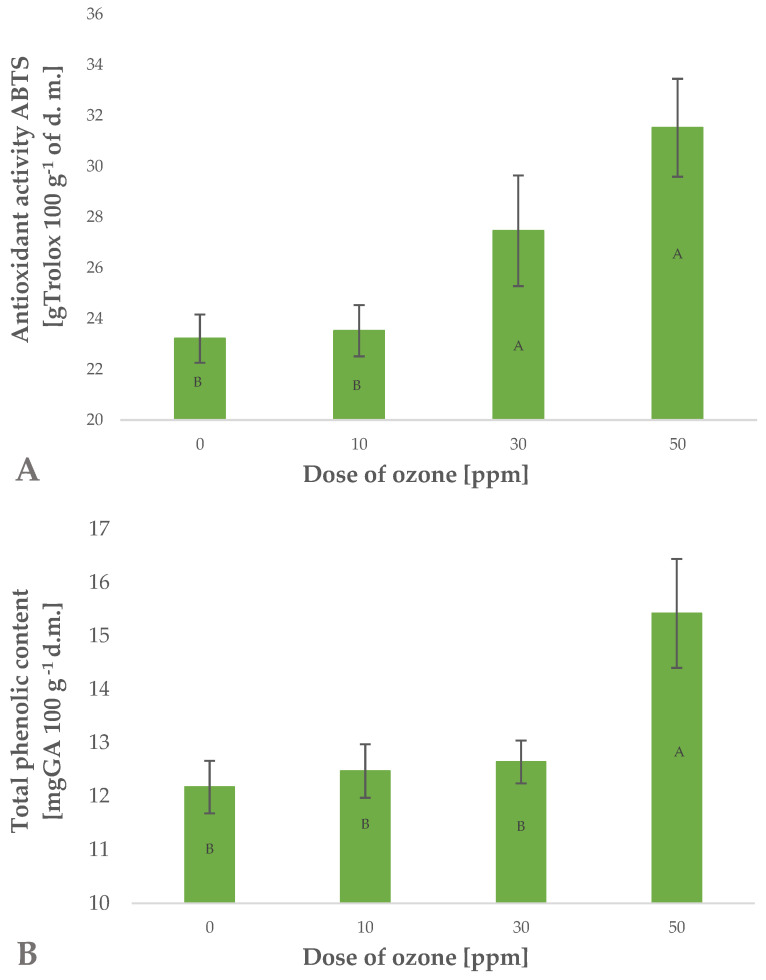
Antioxidant activity ABTS (2,2′ -Azino-bis-(3-ethylbenzothiazoline-6-sulfonic acid) test (**A**) and total polyphenolic content (**B**) in barley young plants depending on the dose of ozone (*n* = 20): differences between the results for the dose of ozone treatment are marked with capital letters; significance level is defined as *p* < 0.05.

Ozone treatment applied to barley plants affected the total content of polyphenols in the samples (Figure 2B). The effect was significantly related to the dose of gaseous ozone used. This is reflected by Pearson’s correlation coefficient of 0.8486. It was found that the highest dose of gaseous O_3_ (50 ppm for 10 min) produced a significant increase in total polyphenols in raw young barley plants. This content was 26.7% higher than in the control sample (not exposed to ozone), while a similar chlorophyll content (32–37 SPAD) identified in barley leaves showed that the applied dose produced no phytotoxic effects. It should be noted that plants exhibit varied tolerances to the effects of gaseous O_3_. The anticipated high tolerance of young plants of *Hordeum vulgare* L. to gaseous O_3_ possibly results from the species-specific characteristics. This was shown by Temple et al. [26], who observed no damage to plants at a twice tropospheric concentration of O_3_. Other young green plants, such as marjoram (*Origanum majorana* L.) and red-veined sorrel (*Rumex sanguineus ssp. sanguineus*), seem to be less resistant to this reactive gas. Plants of red-veined sorrel were found to tolerate exposure to ozone at a rate of 1 ppm (mg m^−3^) at the most. Once this dose was exceeded, it was possible to observe phytotoxic effects. However, as in the case of young barley plants, a higher dose of O_3_ led to an increase in the total polyphenolic content of the raw plant material produced [22]. Similar effects of gaseous O_3_ were demonstrated in the cases of marjoram [25] and rocket plant (*Eruca sativa Mill.*) [27].

Polyphenols are known to significantly affect the antioxidant potential of plant materials (Figure 2A). The observed increase in total polyphenols in trioxygen-treated young barley plants directly impacted the antioxidant potential of the raw material produced. This increase was closely related to the applied dose of gaseous O_3_ (Pearson’s correlation coefficient of 0.9538). The 35.8% increase in antioxidant potential observed in samples exposed to gaseous O_3_ at a rate of 50 ppm for 10 min was higher than the increase in total polyphenols. This may be linked to the fact that total antioxidant potential is affected not only by polyphenols but also by a number of other compounds, including small-molecule substances and compounds with greater molecular weight, such as enzymatic systems and selected vitamins. The absence of phytotoxic effects of O_3_ does not prove that the gas fails to penetrate the cell structures in ozone-treated plants. The presence of ozone in cell structures as a rule leads directly to oxidation of these structures or to activation of the plant’s defence mechanisms [28]. The defence mechanisms are involved in generating substances which antagonise the oxidative effects of O_3_ or its breakdown products, such as ROS and other reactive species, most frequently classified in the group of free radical scavengers [29]. These substances are mainly small-molecule antioxidants. Depending on the ozone treatment conditions applied, the findings show different effects of O_3_ on the antioxidant potential of the ozone-treated raw material. In most cases, green plants generate higher quantities of small-molecule antioxidants at relatively low doses of O_3_, as reported by Matłok et al. [22]. On the other hand, fruit and other crops subjected to ozone treatment directly after harvest were also found to have higher antioxidant capacity, however, this increase was produced by significantly higher doses of gaseous O_3_. Zardzewiały et al. [20] demonstrated that ozone treatment applied to rhubarb petioles at a rate of 100 ppm led to a significant increase in the antioxidant potential of the raw material kept in storage. Lower concentrations of O_3_ also affected the antioxidant potential of ozone-treated rhubarb, however, the increase was considerably lower than the change produced by higher O_3_ doses.

**Figure 3:**
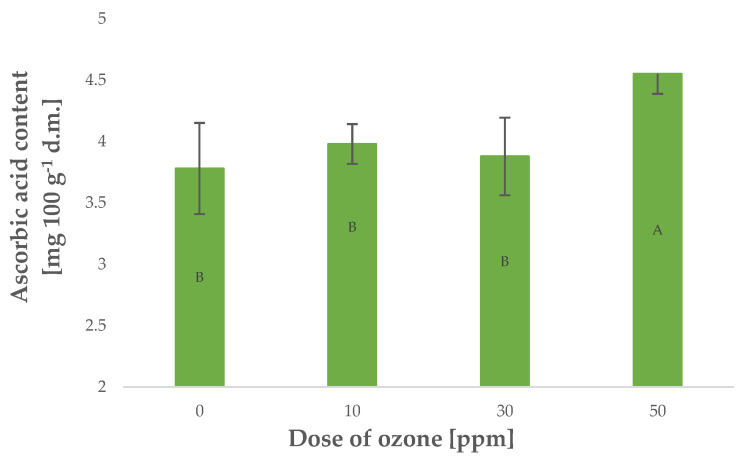
Ascorbic acid content in barley young plants depending on the dose of ozone (*n* = 20): differences between the results for the dose of ozone treatment are marked with capital letters; significance level is defined as *p* < 0.05.

Vitamin C is a highly labile component that is particularly susceptible to oxidising agents such as gaseous O_3_. On the other hand, plants generate larger quantities of this component when exposed to stress [30]. Young barley plants were found with higher contents of vitamin C, corresponding to a higher dose of O_3_ applied in gaseous form Figure 3. The relationship, however, was not as strong as in the case of polyphenol content and antioxidant potential, as reflected by the Pearson’s correlation coefficient of 0.8145 calculated for vitamin C content. Notably, in most cases, vitamin C content decreases in plant materials exposed to gaseous O_3_. This relationship was demonstrated for *Rheum rhaponticum* L. petioles [20], as well as raspberries [31]. Conversely, ozone treatment made it possible to maintain higher contents of vitamin C in raw plant materials kept in storage in an atmosphere of gaseous O_3_.

### 2.2. Determination of Polyphenolic Compounds

Polyphenols are a group of compounds exhibiting high biological activity, which is largely determined by their chemical structure, in particular the type of aglycone and the number and attachment site of the sugar residues [32].

The material investigated in the study was found to contain 16 polyphenolic compounds, irrespective of the ozone treatment conditions (Table 1). The predominant component of the polyphenol mixture identified in all the samples of young barley leaves was isovitexin 7-*O*-glucoside (saponarin) (Figure 3) (79.42% in young barley leaves exposed to O_3_ at a rate of 10 ppm for 10 min, and 82.31% in young barley leaves exposed to O_3_ at a rate of 50 ppm for 10 min). The predominant contents of this compound in young barley leaves was also demonstrated by other researchers. Kamiyama and Shibamoto [8] reported that this compound is the main component of the polyphenol mixture in young green barley leaves and it determines the biological value of the material. Owing to this component, barley leaves are highly suitable as an ingredient in dietary supplements designed to prevent oxidation-induced diseases, especially cancer, inflammation and cardiovascular diseases [8]. It should be emphasised that the conditions applied during the ozone treatment in the present study considerably affected the total polyphenol contents in young barley plants (Figure 2B), consequently leading to proportionally higher contents of isovitexin 7-*O*-glucoside (saponarin) in the material produced.

**Figure 4:**
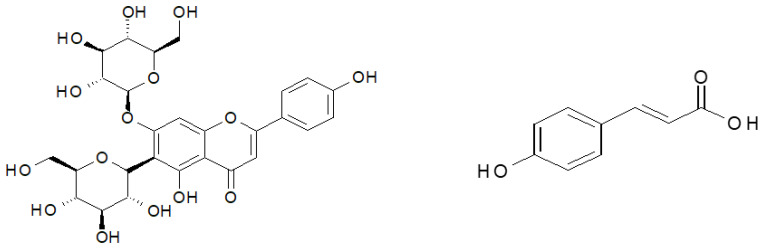
Structures of most represented antioxidant compound. Isovitexin 7-*O*-glucoside. *p*-coumaric acid.

Ozone treatment led to changes in the contents of the specific polyphenolic compounds (Table 1). The significant effect of the ozone treatment was mainly reflected in the increased content of *p*-coumaric acid (Figure 4), with the highest increase (~500% relative to the control sample) identified in young barley leaves exposed to O_3_ at a rate of 30 ppm. The compound has strong antineoplastic properties, particularly effective in reducing the risk of stomach cancer. Furthermore, the presence of this substance in polyphenol mixtures produces strong synergistic effects with other compounds in the group [33]. Considerable differences were also identified in the contents of an unspecified caffeic derivative. During the process of food digestion, an aglycone in the form of caffeic acid is released in the gastrointestinal tract; the compound has a very similar structure to that of *p*-coumaric acid, and as a result, it also exhibits similar biological properties. It is likely that ozonation activates metabolic pathways in which *p*-coumaric acid derivatives, including caffeic acid glycosides, are produced. A similar effect of ozone treatment was observed in the proportional content of feruloyl-caffeic acid, which increased nearly twofold in samples of young barley plants exposed to gaseous O_3_ at a rate of 30 ppm. Notably, the ozone treatment applied to the raw material led to a reduced content of isovitexin derivatives, which also exhibit high biological activity, but due to their high molecular weight, they cross biological membranes with greater difficulty, so their in vivo activity is lower than that of low-molecular weight compounds.

### 2.3. Antioxidant Enzymes Activity

**Figure 5:**
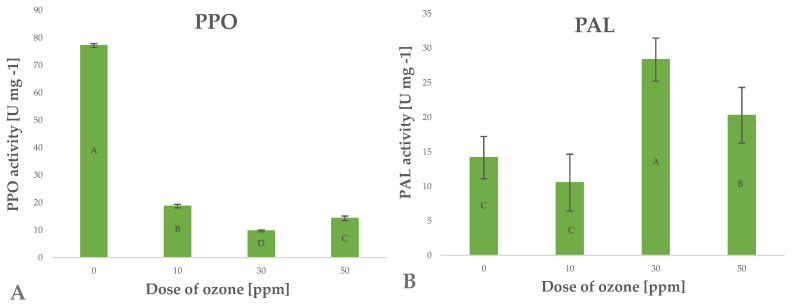
Polyphenol oxidase PPO (**A**) and phenylalanine ammonia lyase PAL (**B**) activity in barley young plants depending on the dose of ozone (*n* = 20): differences between the results for the dose of ozone treatment are marked with capital letters; the significance level is defined as *p* < 0.05.

Polyphenols are secondary metabolites generated in plants for various purposes, such as activation of defence mechanisms, mainly against factors inducing oxidative stress [32]. These factors are generated by enzymatic systems, mainly phenylalanine ammonia-lyase (PAL). The enzyme converts L-phenylalanine to ammonia and trans-cinnamic acid, which triggers the metabolic pathways of many polyphenols. [34]. Research shows that raw plant materials exposed to gaseous ozone respond by altering the activity of this enzyme. Depending on the conditions of the treatment applied, the activity of this enzyme increases significantly, as shown in the case of green peppers subjected to ozone treatment [35]. Piechowiak et al. [31] showed that increased activity of this enzyme directly affected the biosynthesis of polyphenols. This relationship was observed in the case of young barley leaves exposed to ozone (Figure 5B). The highest polyphenol content was found in young barley plants treated with gaseous O_3_ at a rate of 50 ppm for 10 min; in this sample, the findings showed a 43.3% increase in PAL activity, relative to the value identified in the control (untreated) sample.

Polyphenols are degraded in different ways, in processes induced by different oxidative enzymes, including polyphenol oxidase (PPO) [36]. This highly active enzyme promotes the negative effects associated with the production of dark pigments through oxidation of polyphenols to quinones, which then undergo polymerisation [37]. The activity of this enzyme may be inhibited by blanching, change of pH, and other abiotic factors, including O_3_ [38]. In the current study, the conditions applied during the ozone treatment process were significantly (Pearson’s correlation coefficient of 0.8054) related to the decrease in PPO enzyme activity in young barley plants (Figure 5A). The samples treated with the highest dose of ozone were found to have PPO activity that was approximately five times lower than in the control (untreated) samples. The ozone treatment produced a synergistic effect reflected by the simultaneous activation of PAL and inhibition of PPO, which directly led to a considerable increase in the total content of polyphenols in ozone-treated material (Figure 2B). Furthermore, this is correlated with a significant increase in the antioxidant potential of young barley plants (Figure 2A).

### 2.4. Enzymatic Antioxidants

**Figure 6:**
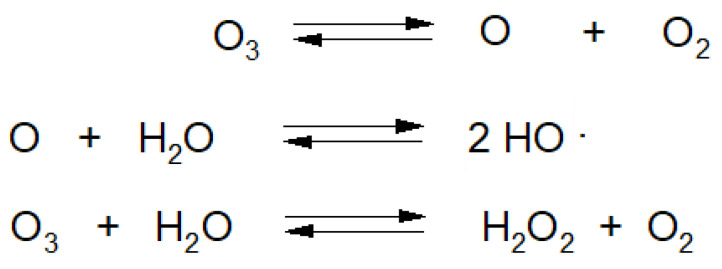
Reactions of ozone decomposition in water.

Majority of raw plant materials consist of more than 90% H_2_O [21]. O_3_ dissolving in H_2_O is decomposed into various types of species, most of which are classified as reactive oxygen species (ROS) (Figure 6) [39].

**Figure 7:**
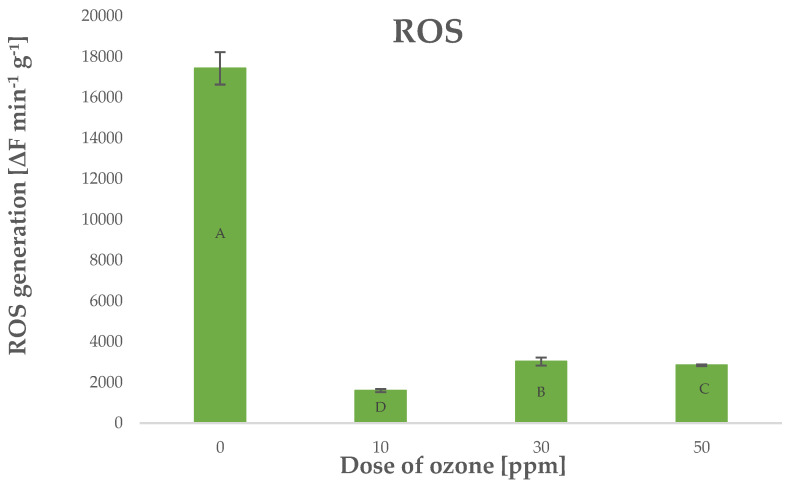
ROS generation in barley young plants depending on the dose of ozone (*n* = 20): differences between the results for the dose of ozone treatment are marked with capital letters; the significance level is defined as *p* < 0.05.

Ozone treatment in such systems should lead to increased production of ROS because O_3_ is highly mobile and it penetrates internal structures of plants exposed to it. Surprisingly, young barley plants treated with O_3_ were found with lower levels of ROS generated in the process. The level of ROS generated in ozone-treated barley plants depended on the dose of O_3_, as shown by Pearson’s correlation coefficient of −0.7288 (Figure 7).

**Figure 8:**
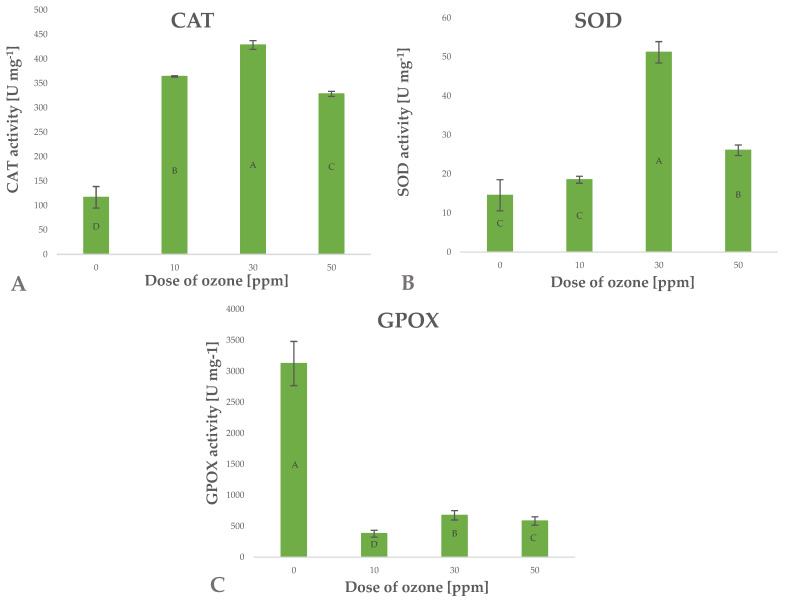
Catalases CAT (**A**), glutathione peroxidases SOD (**B**), guaiacol peroxidase GPOX (**C**) in barley young plants depending on the dose of ozone (*n* = 20): differences between the results for the dose of ozone treatment are marked with capital letters; the significance level is defined as *p* < 0.05.

Antioxidants include a wide range of substances that interfere with the propagation of free radicals and prevent oxidative damage to cell structures. Being a strong oxidant, ozone induces in vitro oxidation of labile components, which usually leads to the loss of antioxidant compounds [40]. The increase in the content of small-molecule antioxidant components observed in the present study may be linked to possible activation of defence mechanisms in the cells of ozone-treated barley plants [40]. Ozone as a gas tends to penetrate biological systems conducting active gas exchange, which results in a cascade of defence responses to oxidative stress. Enzyme systems responsible for scavenging ROS, including H_2_O_2_, are the main defence mechanisms. The effectiveness of this mechanism is reflected by the significant increase in CAT enzyme activity (Figure 8A), directly leading to a decrease in the level of ROS generated in cells of barley plants exposed to gaseous O_3_. The activity of this enzyme depends on the concentration of gaseous O_3_ applied, as reflected by Pearson’s correlation coefficient of 0.6692. Superoxide ions (O_2_^–^) are generated at the time gaseous O_3_ penetrates biological structures with high contents of lignin, H_2_O and sugars [41]. These responses trigger the defence mechanism involving an increase in the activity of the enzyme SOD (superoxide dismutase), which directly breaks down superoxide ions to hydrogen peroxide (H_2_O_2_) and molecular oxygen (O_2_) (Figure 8B). The resulting surplus H_2_O_2_ is broken down by CAT.

The high activity of CAT and SOD enzymes in the ozone-treated barley plants suggests that the ability to reduce stress factors was effectively induced. This observation is consistent with the results showing low levels of ROS generated in cells of ozone-treated plants and decreased activity of guaiacol peroxidase (GPOX) (Figure 8C), which is a known marker of oxidative stress. It appears that the activity of the latter enzyme depends (Pearson’s correlation coefficient—0.7304) on the concentration of gaseous O_3_ applied in the treatment.

Similar relationships between enzymatic activity and ozone treatment conditions were observed in the case of raspberries [42]. This is a typical response of raw plant materials to controlled stress induced by ozone treatment carried out under well-defined conditions.

## 3. Materials and Methods

### 3.1. Plant Materials

The research material comprised young barley (*Hordeum vulgare* L.) plants averaging 10.3 ± 1.8 cm in height.

### 3.2. The Ozone Treatment of the Plant Material

The pots with barley (*Hordeum vulgare* L.) plants (30 pcs.) were placed in the ozone test chamber 10 days after plant emergence. The ozone treatment process was performed in line with the methodology proposed by Matłok et al. [22]. The plants (30 pcs. for all replications) were exposed to gaseous ozone (O_3_) at concentrations of 10, 30 and 50 ppm for 10 min, with 10 l min^−1^ flow rate of ozone. The ozone treatment was carried out at a constant temperature of 25 °C in three replications. The control sample comprised plants which were placed for 10 min in the test chamber with no ozone (concentration of O_3_ = 0.00 ppm). After the ozone treatment was completed, the plants were kept at 25 °C for 24 h to induce biosynthesis of bioactive compounds. After this time, barley leaves were assessed for the content of chlorophyll using the SPAD method [22]; this measurement was performed to determine the potential phytotoxic effects of the ozone gas concentrations applied. Control plants were found with a mean chlorophyll content of 38.7 ± 3.41. The plants exposed to gaseous ozone O_3_ at a rate of 10, 30 and 50 ppm had mean chlorophyll contents of 37.25 ± 3.66; 34.06 ± 2.07; 32.30 ± 2.17, respectively. There were no significant differences in the identified chlorophyll contents of barley leaves relative to the ozone treatment, which shows that the O_3_ concentrations applied produced no phytotoxic effects. Subsequently, the raw material in the form of young barley leaves was collected and subjected to chemical and biochemical analyses to assess a number of factors, including the level of ROS generated, enzyme activity (SOD, CAT, GPOX, PPO, PAL), vitamin C content, total polyphenol content and profile of polyphenolic compounds, as well as antioxidant potential measured using the ABTS assay.

### 3.3. Determination of Antioxidant Activity

Antioxidant activity of *Hordeum vulgare* L. leaves was assayed using ABTS (2.2′-Azino-bis-(3-ethylbenzothiazoline-6-sulfonic acid) methods described by Matłok et al. [14]. The results were expressed as Trolox equivalent [mg] per 100 g of leaf dry matter.

### 3.4. Total Phenolic Content Assay

The level of total phenolic content was measured following the method presented by Matłok et al. [15]. The results were expressed as gallic acid equivalent [mg] per 100 g of leaf dry matter.

### 3.5. Total Ascorbic Assay

Ascorbic acid was extracted from *Hordeum vulgare L.* tissue (2 g) through homogenisation with 10 mL of 2% oxalic acid solution and centrifugation of the homogenate at 10,000× *g* (30 min). The content of ascorbic acid in the supernatant was determined by a titration method, using 2,6-dichlorophenolindophenol according to the methodology described by Piechowiak et al. [31]. The results were converted to 100 g of raw matter.

### 3.6. Polyphenolic Compounds Analysis

#### 3.6.1. Sample Preparation

Young barley plant samples (5 g) designated for the analyses were suspended in a 10 mL solution of 50% MeOH, and then homogenised into a uniform mass. The suspension was then centrifuged to separate any solid residues of the plant material, in line with the methodology presented by Matłok et al. [19].

#### 3.6.2. Determination of Polyphenols Profile

Determination of polyphenolic compounds was carried out using the ultra-performance liquid chromatography (UPLC) Waters ACQUITY system (Waters, Milford, MA, USA) zgodnie z metodyka opisaną przez Matłok et al. 2021 [19].

### 3.7. SOD, CAT, GPOX, PPO and PAL Activity

In order to assess SOD, CAT, GPOX, PPO as well as PAL activity, 1 g samples of refrigerated and milled leaves (−67 °C) were homogenised with 5 mL of 0.9% saline solution containing 0.05% Triton X−100, 1% PVP as well as a protease inhibitor cocktail. The resulting homogenate was then centrifuged at 10,000× *g* for 30 min. (4 °C). SOD activity in the supernatants was measured using the adrenaline test, assessing inhibition of adrenaline auto-oxidation by the dismutases present in the extract. The kinetics of changes in absorbance were measured at a wavelength of 490 nm for 5 min. The unit of SOD activity was defined as the amount of the enzyme inhibiting adrenaline autooxidation by 50%. The CAT activity was assessed by measuring residual hydrogen peroxide in the reaction mixture containing the enzyme; the test applied ammonium metavanadate. The absorbance of the resulting compound was measured at 456 nm. GPOX activity was assessed using a spectrophotometric method enabling measurement of tetra-guaiacol produced, with an absorption maximum at 490 nm. PPO activity was assessed using a calorimetric technique where pyrocatechol was applied as a substrate. The kinetics of changes in absorbance were measured at 420 nm for 30 min. Measurement of PAL activity was based on spectrophotometric assessment of the content of t-cinnamic acid extracted at 290 nm during the reaction of PAL with phenylalanine in an alkaline medium, in line with the method described in a study by Piechowiak and Balawejder [42]. The units of CAT, GPOX, PPO and PAL activity were defined as the amount of protein which produces an increase in absorbance of the reaction mixture by 0.01 in 1 min (CAT, PPO), 1 s (GPOX) and 1 h (PAL), at wavelengths specified in the respective methods. Protein contents in enzyme extracts were assessed using the Bradford method.

### 3.8. ROS Level Analysis

The level of reactive oxygen species in leaf lamina was measured using the fluorimetric method and 2′.7′-dichlorodihydrofluorescein diacetate. The results were expressed as an increase in fluorescence during 1 min in 1 g of tissue [31].

### 3.9. Statistical Analysis

One-dimensional ANOVA analysis of variance of results was conducted at the significance level α = 0.05 utilised STATISTICA 13.1 software (TIBCO Software Inc., Hillview Avenue, Palo Alto, CA, USA). The mean values calculated from the three independent replications were analysed statistically by comparing the results between the variants of the experiment.

## 4. Conclusions

The present study showed that young barley plants are a good source of bioactive compounds. It was also demonstrated that gaseous trioxygen (O_3_) applied in the correct manner induces biosynthesis of compounds, affecting the antioxidant capacity of the ozone-treated plant material. Furthermore, the study assessed the effect of gaseous O_3_ on changes in total polyphenol content and profile, total antioxidant potential and vitamin C content. The highest contents of these compounds were identified in young barley plants treated with gaseous O_3_ at a rate of 50 ppm for 10 min. Saponarin (isovitexin 7-*O*-glucoside) was identified in all the samples of young barley plants as the predominant bioactive component representing polyphenols, accounting for approximately 80% of the contents. On the other hand, the proportional content of the remaining components largely depended on the conditions of the ozone treatment. It was shown that induction of increased biosynthesis of bioactive molecules was directly linked to modification of the activity of selected enzymes. The increased polyphenol content resulted from the modified activity of PPO and PAL. On the other hand, the oxidative effect of ozone on barley plants was reduced owing to the modified activity of CAT, SOD, and GPOX, which led to reduced levels of ROS generated. Analysis of the results showed that by applying gaseous O3, the contents of bioactive compounds can be increased in a residue-free way by inducing oxidative stress defence mechanisms.

## Figures and Tables

**Table 1:** Individual phenolic compounds identified by UPLC-PDA-MS/MS in barley young plants.

Compound		RT *	λ_max_	[M-H] m/z		Content (%)			
		min	nm	MS	MS/MS	0 ppm 0 min	10 ppm 10 min	30 ppm 10 min	50 ppm 10 min
1	*p*-coumaric acid	2.73	309	163	119	0.06	0.30	0.34	0.25
2	Caftaric acid	2.96	288 sh. 327	311	179	0.15	0.19	0.30	0.17
3	Feruloyl-caffeic acid	3.15	288 sh. 322	367	193	0.12	0.14	0.21	0.09
4	Unspecified caffeic derivative	3.24	288 sh. 324	425	367	0.04	0.31	0.34	0.32
5	Luteolin 6-*C*-arabinoside-8-*C*-glucoside	3.38	274. 341	579	447. 285	1.25	0.92	0.90	0.91
6	Isoscoparin 7-*O*-glucoside	3.51	276. 333	623	461. 299	0.22	0.32	0.36	0.04
7	Isoorientin 7-*O*-glucoside	3.75	269. 347	609	447. 285	1.21	1.18	0.91	1.42
8	Isovitexin 7-*O*-glucoside	3.88	269. 331	593	431	81.72	79.42	82.06	82.31
9	Isovitexin 7-*O*-(6″-*p*-coumaroyl)-glucoside	3.98	271. 324	739	593	1.49	2.69	1.82	2.05
10	Isovitexin	4.18	270. 338	431	269	0.52	0.03	0.20	0.35
11	Isoorientin 7-*O*-(6″-sinapoyl)-glucoside	4.57	271. 338	815	447	0.43	0.25	0.37	0.22
12	Isoorientin 7-*O*-(6″-feruloyl)-glucoside	4.72	271. 338	785	447	0.40	0.05	0.09	0.12
13	Isovitexin 7-*O*-(6″-sinapoyl)-glucoside	5.00	271. 334	799	431	6.33	8.86	7.42	6.61
14	Isovitexin 7-*O*-(6″-sinapoyl)-glucoside-4′-*O*-glucoside	5.11	270. 338	961	799. 593	0.51	0.37	0.18	0.11
15	Isovitexin 7-*O*-(6″-feruloyl)-glucoside	5.21	271. 331	769	431	4.71	4.48	4.04	4.50
16	Apigenin 6-*C*-arabinoside-8-*C*-glucoside	5.35	269. 329	563	443	0.87	0.51	0.47	0.54

* RT—retention time; sh = shoulder peak; ±SD and *n* = 3; FD. Note. Differences in results between the dose of ozone are indicated by different capital letters. difference at significant level α < 0.05.

## Data Availability

The data presented in this study are available in this article.
